# Depressive symptoms, parental stress, and attachment style in mothers and fathers two and a half years after childbirth: Are fathers as affected as mothers?

**DOI:** 10.1177/1367493520942050

**Published:** 2020-07-15

**Authors:** Maude Johansson, Thomas Nordström, Idor Svensson

**Affiliations:** 1 Department of Psychology, 427813 Linnaeus University, Sweden

**Keywords:** Attachment style, depressive symptoms, mothers and fathers, parental stress

## Abstract

The study aimed to determine the prevalence of depressive symptoms and whether parental stress and attachment style affected depression in mothers and fathers two and a half years after the birth of a child. The parents completed several questionnaires including the Edinburgh Postnatal Depression Scale, the Swedish Parenthood Stress Questionnaire and the Relationship Questionnaire. The prevalence rate of depressive symptoms in mothers was 14.9%, while for fathers it was 11.5%. Differences between the parents identified as depressed and those without depressive symptoms were also analysed. There were no significant differences between depressed and non-depressed parents on the secure, avoidant and fearful attachment styles. However, there was a significant difference between groups on the preoccupied subscale. The final aim was to calculate if attachment style contributed to the level of depression while accounting for the impact of parental stress. Parental stress (incompetence, social isolation and spouse relationship problems) was the best predictor for mothers’ depressive symptoms, while parental stress (social isolation and health) and the preoccupied attachment style were the best predictors for such symptoms in fathers. The findings indicated that parental stress and depressive symptoms are closely related and can explain the difficulties parents face.

## Introduction

Contemporary research has found that post-partum depression in mothers is common, with a prevalence rate ranging from 10% to 20%, depending on the criteria used for the diagnosis (Brummelte and Galea, 2016; Gavin et al., 2005). Meanwhile, post-partum depression symptoms are evident in between 5% and 12% of fathers (Paulson et al., 2006; Paulson and Bazemore, 2010; Philpott and Corcoran, 2018). The appearance of these symptoms in fathers is also found to be closely related to maternal post-partum depression (Schumacher et al., 2008).

Studies on depression following delivery have primarily focused on mothers, generally in the first year after birth. However, in a study conducted in a Swedish county, the prevalence of depressive symptoms was found to be 11.3% for mothers and 4.9% for fathers (Johansson et al., 2017). Several research studies additionally show that parents’ well-being and depressive symptoms may be even higher after the first year post-partum (Josefsson et al., 2001; Magnusson et al., 2011; Paulson and Bazemore, 2010; Woolhouse et al., 2015). Moreover, it was found that women experiencing depression during pregnancy are approximately six times more likely to experience depression later in life than women who do not experience the illness during this period (Josefsson and Sydsjö, 2007).

Depressive symptoms and parental stress are also prevalent in fathers. Studies show that fathers as well as mothers are affected by the same types of mood alteration during the transition to parenthood and that their mental health has a substantial impact on the child’s development and the health of the family (Goodman, 2004; Ramchandani et al., 2005). Post-partum depression, especially in fathers, has been noted to exacerbate the effects of maternal depression on a child’s behavioural problems (Mazzeschi et al., 2015; Paulson, et al., 2006). Parents can expect post-partum depression as well as experiencing parental stress after delivery. While most parents experience some degree of parental stress, in some cases, this experience is expressed in strong feelings of incompetence in parenthood (Östberg et al., 1997), spouse relationship problems (Johansson et al., 2017; Kerstis et al., 2012; Lavee et al., 1996), attachment problems and difficulties in parenting behaviour (Sroufe et al., 2010). Research has also established a link between post-partum depression and parental stress, concluding that post-partum depression is the most reliable predictor for parental stress (Leigh and Milgrom, 2008). Post-partum depression and depressive symptoms in parents as well as parental stress during a child’s infant and toddler years can therefore harm the child’s development, as the parent experiencing depression often has difficulty interacting and being emotionally accessible (Goodman et al., 2011).

### Attachment style

According to Bowlby, interactions with the parents (attachment persons) during infancy and childhood are important, as these relationships give rise to an inner working model of relationships, creating an individual’s attachment style which guides all attachment-related cognitions, affectations and behaviour from infancy to old age (Bowlby, 1969). Attachment style has an impact on later relationships throughout the lifespan, including relationships with friends and romantic partners, as well as with children (Simpson and Rholes, 2012).

Ainsworth invented a methodology (the strange situation) from Bowlby’s theory, which made it possible to test Bowlby’s ideas empirically and also expanded the theory (Ainsworth and Bowlby, 1991). Ainsworth contributed the concept of a secure base from which the child can explore its surroundings and develop maternal sensitivity, which is the caregiver’s sensitivity to signals from the infant. Bowlby observed that children’s attachment style is established from their interactions with their caregivers. Parental accessibility or inaccessibility contributes to the development of the child’s attachment style. If the parents are accessible and helpful, then these expectations develop the child’s attachment style. Ainsworth and Bowlby (1991) have identified four attachment styles (secure, avoidant, preoccupied and fearful). A person with an avoidant attachment style is characterised by the need to have relationships with others, at the same time experiencing discomfort in close relationships and worrying about the potential for being hurt. A person with a preoccupied attachment style seeks high levels of intimacy, approval and responsiveness, sometimes to such an extent that they become overly dependent on the attachment figure. A fearful-avoidant attachment style can develop when a person has been exposed to losses or trauma, such as sexual abuse in childhood and adolescence.

The descriptions of adult attachment styles offered below are based on the Relationship Questionnaire (RQ) devised by Griffin and Bartholomew (1994) which was used in this study. Research has shown that insecurely attached people are more prone to depressive symptoms (George et al., 1996) and that attachment style is a predictor of parental stress three months after birth (Mazzeschi et al., 2015). Depressive symptoms and parental stress are already well-researched concepts during parents’ first year after delivery, at least for mothers. Research has also held that depression has a huge impact on parents’ ability to attach emotionally to the child, which is an important aspect of the child’s development (Bifulco et al., 2002).

### Aims

The aims of this study were to examine the prevalence of depressive symptoms and to ascertain how previously identified risk factors, such as parental stress and attachment style, impact the depressive symptoms in mothers and fathers two and a half years after the birth of a child. To this end, the following research questions were determined:

1. What is the prevalence of depressive symptoms in mothers and fathers two and a half years after birth, and what is the association between depressive symptoms and parental stress?
2. Is there a connection between attachment style and depressive symptoms?
3. Does attachment style contribute to the level of depression while accounting for the impact of parental stress?

## Methods

### Participants

The inclusion criteria for being eligible to enter the study were parents visiting the Child healthcare centre (CHC) at their mandatory two-and-a-half-year medical control. This control is part of the Swedish healthcare, free of charge. In principle, all parents in the country visit it regularly, for parental support and child healthcare. As part of the planned visit, when the child has reached the age of two and a half years, parents were asked by their nurse to participate in the study. The nurses had good knowledge of the parents from previous visits to the centre. On this occasion, the nurses asked the parents to participate in the study by completing the questionnaire at home. The couples were asked to complete the questionnaire independently of each other and to post them separately to the researchers. Parents not fluent in Swedish, as assessed by their nurse, were not included in the study. Recruiting continued until no more parents responded favorably to entering the study.

### Instruments

#### Depressive symptoms

The Edinburgh Postnatal Depression Scale (EPDS) is a ten-item screening scale used in the CHC to identify post-partum depressive symptoms. This scale has been validated on both mothers (Wickberg and Hwang, 1996) and fathers (Massoudi et al., 2013), where a cut-off score of 12 points or more for indicating depressive symptoms has been suggested. In the present study, the Cronbach alpha for the EPDS was .67.

#### Parental stress

The Swedish Parenthood Stress Questionnaire (SPSQ) includes questions measuring different aspects of perceptions of stress in the parental role. It includes questions concerning the overall experience of caregiving feelings within the parental role and statements about parenthood difficulties. The SPSQ items use a 5-point Likert scale, which involves five subscales: feelings of incompetence, spouse relationship problems, social isolation, health problems and restrictions of the role. The SPSQ is stable with both non-clinical and clinical Swedish samples (Östberg et al., 1997). The Cronbach alpha for the different subscales was calculated in this study as follows: incompetence, .72; spouse relationship problems, .67; social isolation, .64; health problems, .63 and role diffusion, .78.

#### Attachment style

The RQ was included in order to examine the parents’ attachment. This questionnaire is an instrument for measuring adult attachment styles (Griffin and Bartholomew, 1994) involving four categories—secure, avoidant, preoccupied and fearful styles. Each question is scored on a 7-point Likert scale. The RQ has been widely used in many areas involving relationship research, especially before the year 2000. For more details, see the review by Crowell et al. (1999). In most studies measuring attachment style, reliability estimation has not been reported. However, in one study, a Cronbach’s alpha of .77 was reported (Beck and Madresh 2008).

### Statistical analyses

For the first aim, prevalence of depression was calculated in terms of cut-off and percentages for both mothers and fathers. The relationship between depressive symptoms and parental stress was estimated using Pearson’s product–moment correlation coefficient for both mothers and fathers. Frequencies and percentages were used to estimate how many parents had had treatment, the type of treatment they had received and whether the treatment had been successful.

For the second aim, Mann–Whitney U tests were used to estimate differences on each RQ subscale between depressed and non-depressed parents. As four estimates of differences were conducted, Bonferroni adjustments with Holm’s correction were applied in order to keep the family-wise error rate at 5%, while maintaining a reasonable power to detect actual differences between groups.

For the third aim, standard multiple regression analyses were conducted for mothers and fathers separately. Only the RQ subscales associated with depressive symptoms were included. Finally, in order to rule out any potential confounders (i.e. any influence from the demographics/background factors), we correlated such factors with the depression scores.

### Ethical approval

The study received ethical approval from the Regional Ethics Review Board in Linköping, Sweden (Register number 2016/193-31), and informed consent was obtained from the participants.

## Results

The recruiting method resulted in 174 mothers (54%) and 148 fathers (46%), including 127 couples, all of whom answered the questionnaire. Only a few parents were assessed by their nurse as not being fluent in Swedish.

The following demographic variables were collected: gender, age, living or not living with the child’s other parent, working time and education level, as well as the number of children. The mean age for mothers were 32.9 years (SD = 5.5) and 34.6 years (SD = 4.8) for fathers. Ninety-four percent (*n* = 304) of the parents were living with the child’s other parent. Fifty-three percent (*n* = 172) had two children, 30% (*n* = 98) had one child, 12% (*n* = 38) had three children and 6% (*n* = 19) had one or two step-children. In addition, 63% (*n* = 78) of the fathers had an elementary school/secondary school education and 46% (*n* = 69) had a university education. For the mothers, 37% (*n* = 65) had an elementary school/upper secondary school education and 62% (*n* = 109) had a university education.

Most of the parents in the study were working full-time after parental leave (18 months of full pay being provided in Sweden). Of the mothers, 55% (*n* = 94) worked full-time, 36% (*n* = 62) part-time and 9% (*n* = 15) more than full-time. Of the fathers, 72% (*n* = 106) worked full-time, 16% (*n* = 24) part-time and 11% (*n* = 17) more than full-time. Statistical calculations (not reported here) of potentially confounding factors, i.e. demographic factors, background factors and number of children, were not associated with parents’ depressive symptoms or parental stress.

### Prevalence of depressive symptoms

The first aim was to calculate the prevalence of depressive symptoms in both mothers and fathers. Of the mothers, 14.9% had depressive symptoms (EPDS ≥ 12), while for the fathers, the number was 11.5%. The next step was to investigate the relationship between parental stress SPSQ and depressive symptoms EPDS, and, as expected, the relationship was found to be moderate-to-strong between the two variables for fathers (*r* = .42, *n* = 144, *p* < .001) and for mothers (*r* = .51, *n* = 171, *p* < .001).

### Received treatment

Within the sample, 60 (18.6%) of the parents had received treatment for depression. Of these, five parents had obtained medication. Thirty-five parents had received counselling/psychotherapy and 19 parents both counselling and medication. Fifteen of the parents believed that the medication had been helpful, while nine parents did not. In addition, 37 parents considered the counselling/psychotherapy to be helpful, while 15 parents had not received any help from counselling or psychotherapy for their depression.

### Depression and attachment style

The second aim was to investigate whether there were any differences between the parents identified as depressed and those without depressive symptoms on the RQ subscales. The analyses are summarised in Table 1. Neither the secure, avoidant or fearful subscale scores (tested for differences using the median with interquartile ranges) were significantly different between depressed and non-depressed parents, when controlling for multiple testing. However, there was a significant difference between groups on the preoccupied subscale (adjusted *p* = .028), where the median for depressed parents was three points (25th–75th percentile = 2–5), while non-depressed parents had a median of two points (25th–75th = 1–4) on the scale.

**Table 1.** Attachment style and post-partum depression classification (depressed and non-depressed) in mothers and fathers combined 2.5 years after birth.

	Secure					Avoidant					Preoccupied					Fearful				
	M	25th–75th	IQR	*p*-value	Adj. *p*-value	M	25th–75th	IQR	*p*-value	Adj. *p*-value	M	25th–75th	IQR	*p*-value	Adj. *p*-value	M	25th–75th	IQR	*p*-value	Adj. *p*-value
Non-depressed (*N* = 267)	5	3–6	3	.985	1.0	4	2–6	4	.721	1.0	2	1–4	3	.007	**.028**	2	1–3	2	.051	.153
Depressed (*N* = 41)	5	3–6	3			3	2–6	4			3	2–5	3			2	1–5	4		

*Note*: M: median; IQR: interquartile range. The table contains interquartile details (25th–75th percentile and IQR). Mann–Whitney U tests were used to test differences between depressed and non-depressed on each subscale, which is represented by original *p*-values (*p*) and adjusted *p*-values (adj. *p*) from Bonferroni adjustments for multiple testing with Holm’s correction. The adjusted *p*-values are compared against *α* = .05.

### Stress, attachment style and depression

The third aim was to estimate the contribution of RQ subscale scores on depression (using the total score on the depression scale), while accounting for parental stress. As stated in the method section, only those RQ subscales that were found to be associated with depression were included (cf. the second aim). Therefore, only the preoccupied subscale was subject to analysis. In addition, all parental stress subscales were entered in the analyses (no violation of the statistical assumptions of using multiple regression, such as multicollinearity being found). The analyses were conducted separately for mothers and fathers to enable detecting unique predictors for each group. Both analyses are summarised in Table 2.

**Table 2.** Multiple regression analyses with stress subscales and the preoccupied attachment scale as predictors and depression scores as outcome.

	Mothers				Fathers			
	B	*B*	*p*-value	*R*^2^	B	*B*	*p*-value	*R*^2^
Constant	−2.00			.35	−1.44			.26
Incompetence	.203	.359	**<.001**		.076	.135	.140	
Spouse relationship problems	.162	.178	**.028**		.008	.008	.929	
Health problems	.090	.082	.264		.181	.177	**.041**	
Role restriction	−.081	−.127	.149		−.003	−.006	.944	
Social isolation	.210	.277	**.001**		.204	.257	**.003**	
Preoccupied	−.087	−.043	.544		.381	.190	**.022**	

*Note*: Regression model for mothers, *F*(6, 155) = 136.81, *p* ≤ .001. Regression model for fathers, *F*(6, 131) = 7.86, *p* ≤ .001.

For the mothers, the parental stress incompetence subscale contributed the most to the variation in depression scores (St. Beta = .359, *p* < .001), followed by social isolation (St. Beta = .277, *p* = .001) and spouse relationship problems (St. Beta = .178, *p* = .028). The remaining predictors in the model turned out to be non-significant. The model for mothers explained 35% of the variation in depression scores (*R*^2^ = .35). For fathers, social isolation contributed the most to the variation in depression scores (St. Beta = .257, *p* = .003), followed by the preoccupied attachment style (St. Beta = .190, *p* = .022) and health problems (St. Beta = .177, *p* = .041), while the remaining predictors were non-significant. The model for fathers explained 26% of the variation in depression scores (*R*^2^ = .26). In conclusion, parental stress-associated subscales were the best predictors for mothers’ depression scores, while parental stress-associated subscales and the preoccupied attachment style were the best ones for fathers’ depression scores.

## Discussion

This study produced four principal findings. Firstly, the prevalence of depressive symptoms revealed that almost 15% of mothers and 11.5% of fathers scored ≥12 on the EPDS two and a half years after childbirth. Secondly, the association between depressive symptoms and parental stress was moderate for fathers and strong for mothers. Thirdly, of the RQ subscales, only the preoccupied attachment style was associated with depression, as shown by the difference in scores between depressed and non-depressed parents, while no differences between the groups were found on the remaining subscales (i.e. secure, avoidant or fearful). Fourthly, for the mothers, parental stress in the areas of feelings of incompetence, social isolation and spouse relationship problems were the best predictors of depressive symptoms. For the fathers, parental stress in the areas of social isolation and health problems, as well as the preoccupied attachment style, were the best predictors. Both mothers’ and fathers’ depressive symptoms were associated with social isolation; mothers’ symptoms were associated with problems concerning the relationship with the spouse. Health problems were, on the other hand, only associated with fathers’ depressive symptoms.

The findings related to depression rates in the present study were different in comparison with a previous study conducted 25 months after childbirth in the same county. In that study, only 4.9% of the fathers and 11.3% of the mothers experienced depressive symptoms (Johansson et al., 2017). The results of the studies suggest that depressive symptoms do not decrease following the child’s first year after birth.

We also hypothesised that depressive symptoms later in parenthood could indicate that the predictor explaining these parents’ depressive symptoms was an insecure attachment style (including avoidant, preoccupied and fearful). The connection between depressive symptoms and attachment style was not as strong as that between depressive symptoms and parental stress. The hypothesis concerning insecure attachment and depression could therefore not be verified in our study, other than for the preoccupied style, which means that our results were not fully in line with previous research that connects insecure attachment with depression (Bifulco et al., 2002; Murphy and Bate, 1997). The finding of the connection between preoccupied attachment and depressive symptoms needs to be further investigated for both mothers and fathers with depressive symptoms in order to better understand this connection.

Another result was that mothers and fathers experienced parental stress in different areas. Mothers expressed parental stress in feelings of incompetence, social isolation and spouse relationship problems, while fathers experienced parental stress in social isolation and health. The model explained 35% of the variation in depression scores for mothers and 26% for fathers. Social isolation constituted the largest contribution to variation in depression scores, followed by the preoccupied attachment style and health problems in fathers. There was a stronger relationship between parental stress and depressive symptoms for mothers than for fathers, as also found by Widarsson et al. (2013).

Leigh and Milgrom (2008) established a link between post-partum depression and parental stress, concluding that post-partum depression was the most reliable predictor of parental stress. In our study, we propose the opposite hypothesis that parental stress affects depression. Earlier studies have shown that fathers had lower prevalence of depressive symptoms and experienced parental stress in different areas than mothers (Massoudi et al., 2016; Widarsson et al., 2013). Our findings were that fathers’ depressive symptoms are nearly in line with mothers’ depression scores 30 months after delivery. One interpretation of the results can be that changing parental roles in fathers, as more involved in the child’s upbringing with more responsibilities and parental requirements seems to increase the fathers’ depressive symptoms later in parenthood. ‘Work–life stress’ (Bauer et al., 2012) together with ‘work–family conflict’ (Lavee et al., 1996) can constitute two factors that explain the results. Later, in parenthood (30 months after childbirth), most parents are working, often full-time. Requirements from work and the family need to be negotiated to find a solution adapted to the demands of work, the spouse’s and the child’s needs.

Both mothers and fathers experienced parental stress in the form of social isolation (including lack of support or additional requirements from the spouse, relatives, friends and work). If parents lack support during the child’s toddler years, then this often affects ‘work–life stress’ while ‘work–family conflict’ contributes to spouse relationship problems. Studies about ‘work–life stress’ and ‘work–family conflict’ have indicated that these factors are associated with less favourable family characteristics and parental behaviours (Bauer et al., 2012) as well as being related to high levels of depression and poor physical health (Frone et al., 1997). Our research findings indicate that parental stress and depressive symptoms are closely related and that there are different stressors explaining depressive symptoms. Furthermore, from this study, it seems that fathers are affected nearly as much as mothers.

### Implications for practice

EPDS is a well-established screening instrument in the Swedish CHC. While the CHCs screen mothers for depression six to eight weeks after the birth of a child, using a cut-off EPDS ≥ 12, they appear to have failed to identify depressive symptoms later in parenthood. The present study indicates that such symptoms are still prevalent after the first year post-partum. In our study, 58 parents (20%) scored between 9 and 11. These are parents who may develop depressive symptoms later in the child’s preschool years, which is similar to the findings of Magnusson et al. (2011). Skoog et al. (2019) found that post-partum depression in immigrant mothers was difficult to identify by screening. These findings indicate that there is a need to reconsider current models of maternal health surveillance and primary care support. Primary care services need to remain attentive to the high prevalence of maternal depression among women with preschool-aged children by targeting resources to those at higher risk of mental health issues and focusing more on fathers. These findings further indicate that there is a need for a systematic approach to supporting parents, which would involve focusing on both parents.

### Limitations

As the EPDS was constructed and validated for the first year post-partum, the questions may be less useful for identifying the depressive symptoms that occur later. It is also well known that self-reporting measures may be problematic, since the respondents, for social desirability reasons, may underestimate their symptoms and problems, which may in turn affect the measurement. In this study, we used a cut-off of EPDS ≥ 12, which means that, when measurement errors are considered, we risk misclassifying participants who score close to the cut-off. Furthermore, the RQ scale, although widely used in research, may suffer from the same limitation, because the published literature lacks details of reliability and validity estimates. We found only one study, by Bäckström and Holmes (2007), that provided details of the reliability of the scale. Therefore, the conclusions of this study must be considered with caution in addition to the circumstance that the study is correlational and may warrant further investigations.

## Conclusions

The main findings in the study were that depressive symptoms in fathers and mothers are not limited to the first year post-partum. Our findings indicated elevated levels of depressive symptoms in early parenthood beyond the first year of the child’s life for both mothers and fathers. An association was also found between depressive symptoms and parental stress in both mothers and fathers. In regard to mothers’ parental stress, stress-associated subscales were the best predictors for their depression score, while for fathers, depression levels were predicted by parental stress and the preoccupied attachment subscale.
